# Old Parasitoids for New Mealybugs: Host Location Behavior and Parasitization Efficacy of *Anagyrus* *vladimiri* on *Pseudococcus* *comstocki*

**DOI:** 10.3390/insects12030257

**Published:** 2021-03-18

**Authors:** Renato Ricciardi, Valeria Zeni, Davide Michelotti, Filippo Di Giovanni, Francesca Cosci, Angelo Canale, Lian-Sheng Zang, Andrea Lucchi, Giovanni Benelli

**Affiliations:** 1 Department of Agriculture, Food and Environment, University of Pisa, via del Borghetto 80, 56124 Pisa, Italy; renato_ricciardi@hotmail.it (R.R.); d.michelotti1@studenti.unipi.it (D.M.); aphelocheirus@gmail.com (F.D.G.); francesca.cosci1@virgilio.it (F.C.); angelo.canale@unipi.it (A.C.); andrea.lucchi@unipi.it (A.L.); 2 Key Laboratory of Green Pesticide and Agricultural Bioengineering, Guizhou University, Guiyang 550025, China; valeriazeni93@gmail.com (V.Z.); lsz0415@163.com (L.-S.Z.)

**Keywords:** biological control, Encyrtidae, parasitization behavior, parasitoid fitness, *Planococcusficus*, Pseudococcidae

## Abstract

**Simple Summary:**

*Anagyrus vladimiri* has been widely employed as a biological control agent (BCA) against the vine mealybugs *Planococcus ficus* but the knowledge about its employment against other mealybug species is limited. In this study, we investigated the potential efficacy of *A. vladimiri* for *Pseudococcus comstocki* management, considering the increasing threat represented by this mealybug pest in Mediterranean vineyards and fruit orchards. No-choice and two-choice tests were conducted to quantify parasitoid behavior against *P. ficus* and *P. comstocki*. Our results pointed out that *A. vladimiri* successfully parasitized both pests, showing no host preference between the two species. Our observations highlight that this parasitoid can be successfully deployed as BCA against *P. comstocki* populations.

**Abstract:**

The Comstock mealybug, *Pseudococcus comstocki* (Hemiptera: Pseudococcidae) is a primary pest of orchards in the North and Northwest of China. This pest appeared recently in Europe, including Italy, where it is infesting mainly vineyards as well as apple and pear orchards. The present study investigated the efficacy of *Anagyrus vladimiri*, a known biological control agent (BCA) of *Planococcus ficus*, on *P. comstocki* to evaluate a potential use for the management of this new pest. No-choice tests were conducted to quantify the parasitoid behavior against *P. ficus* and *P. comstocki*. The parasitoid successfully parasitized both species (parasitization rate: 51% and 67% on *P. comstocki* and *P. ficus*, respectively). The *A.* *vladimiri* developmental time (19.67 ± 1.12 vs. 19.70 ± 1.07 days), sex ratio (1.16 ± 1.12 vs. 1.58 ± 1.07) and hind tibia length of the progeny showed no differences when *P*. *comstocki* and *P*. *ficus,* respectively, were exploited as hosts. Two-choice tests, conducted by providing the parasitoid with a mixed population of *P. ficus* and *P. comstocki*, showed no host preference for either of the two mealybug species (23 vs. 27 first choices on *P*. *comstocki* and *P*. *ficus*, respectively). The parasitization rate (61.5% and 64.5% in *P. comstocki* and *P. ficus*, respectively) did not differ between the two hosts. Overall, our study adds basic knowledge on parasitoid behavior and host preferences and confirms the use of this economically important encyrtid species as an effective BCA against the invasive Comstock mealybug.

## 1. Introduction

The Comstock mealybug, *Pseudococcus comstocki* (Kuwana) (Hemiptera: Pseudococcidae), is widely recognized as an important insect pest of fruit trees, especially pear trees, in many fruit-producing regions of the world, with special reference to North and Northwest China [1]. Its importance as an emerging pest of fruit, vineyard and ornamental crops in European countries, including Italy, is increasing [2,3] (Parrilli M. and Burgio G, unpubl. data). The nymphs and adult females strongly inhibit the growth and morphogenesis of fruit trees, mainly by feeding on buds, twigs, leaves, fruits, and rootlets, resulting in twig and shoot swelling, longitudinal cracking, abnormal fruit development and production of a large quantity of honeydew, thus causing major economic losses [1,4]. Several studies have been conducted to increase the knowledge about the biology of this mealybug pest, looking for effective control strategies [5,6]. Xu et al. [7] investigated the influence of temperature on *P. comstocki* population, highlighting that 26 °C was the optimal temperature for population growth while low (below 17 °C) and high (above 29 °C) temperatures reduced the population growth rate. Jeon et al. [8] also investigated the incorporation of temperature in the development of forecasting models for timing insecticide applications against *P. comstocki*.

Recently, increasing attention has been given to finding effective and ecologically acceptable methods for controlling Comstock mealybugs. Within the Integrated Pest Management (IPM) scenario, biological control agents (BCAs) such as predatory coccinellid beetles, e.g., *Cryptolaemus montrouzieri* (Coleoptera: Coccinellidae) [9] as well as parasitic Hymenoptera, could provide useful tools. In this framework, Malausa et al. [10] evaluated *Allotropa burrelli* Muesebeck (Hymenoptera: Platygastridae) and *Acerophagus malinus* (Gahan) (Hymenoptera: Encyrtidae) for the control of *P. comstocki* in France. An earlier study aimed at defining the complex of *P*. *comstocki* parasitoids in Italy includes two other species, *Acerophagus maculipennis* (Mercet) (Hymenoptera: Encyrtidae) and *Anagyrus* sp. near *pseudococci* (Girault) (Hymenoptera: Encyrtidae), recently re-described as *Anagyrus vladimiri* Triapitsyn [4,11]. The latter is widely known for its efficacy against other important mealybug species, such as the grapevine mealybug, *Planococcus ficus* (Signoret) (Hemiptera: Pseudococcidae), which recently led to its wide-scale adoption in more than 800 hectares of high-valued organic vineyards (i.e., Bolgheri area, Tuscany, Italy) [12].

Despite the earlier records of *A*. *vladimiri* parasitization on *P*. *comstocki*, no quantitative data are available to shed light on the effectiveness of this encyrtid against the Comstock mealybug. To the best of our knowledge, little is known about *A. vladimiri* host preferences and suitability when foraging on *P*. *comstocki* mixed with other mealybug species, such as the grapevine mealybug. The present study aims to quantify, the oviposition behavior of a mass-reared commercially available strain of *A*. *vladimiri* attacking young females of *P*. *comstocki*. Furthermore, host preferences [13] of *A*. *vladimiri* for the above-mentioned mealybug species over its potential ”optimal” host *P*. *ficus*, were evaluated, both in no-choice and two-choice conditions. The host suitability of the Comstock mealybug for the successful development of *A. vladimiri* was assessed, shedding light on the potential of this encyrtid species for real-world biocontrol attempts.

## 2. Materials and Methods

### 2.1. Insect Rearing and General Observations

Insect rearing and experimental assays were conducted in laboratory conditions at 23 ± 1 °C, 45 ± 5% RH and a 14:10 (L:D) photoperiod. A commercial strain of *A*. *vladimiri*, as well as its routine host *P. ficus*, were maintained as described by Romano et al. [14]. A field strain of *P*. *comstocki*, originally collected in the Emilia-Romagna region (Central Italy), was reared on potato sprouts, a common food substrate for mealybugs, as detailed by Islam and Copland [15]. The whole rearing apparatus was placed in dark rearing cages (180 × 90 × 90 cm) (Bugdorm^®^, Megaview Science, Taiwan).

All experiments were carried out using 2-5-day-old *A. vladimiri* mated females fed ad libitum with a solution of honey and water (1:1, *w*:*v*) and never exposed to mealybug hosts before testing [15].

### 2.2. Oviposition Behavior, Host Preferences, Host Suitability and Quality of the Parasitoid Progeny

The host-seeking and oviposition behavior of *A*. *vladimiri* was quantified on *P*. *comstocki* young females following the method by Chong and Oetting [16] with a few modifications. The host-seeking and oviposition behavior of *A*. *vladimiri* on *P*. *comstocki* were observed using a new Petri dish for each test (hereafter, the arena, diameter 35 mm) under uniform daylight conditions. Two kinds of experiments were conducted: no-choice and two-choice tests, detailed below.

#### 2.2.1. No-Choice Tests

In the no-choice test, a single *A*. *vladimiri* mated female was provided with 8 *P*. *comstocki* (mean ± SD; length x width: 2.65 ± 0.38 × 1.57 ± 0.29 mm; weight: 0.0012 ± 0.0003 g) or 8 *P*. *ficus* young females (mean ± SD; length x width: 2.95 ± 0.14 × 1.78 ± 0.17 mm; weight: 0.0013 ± 0.0002 g). To easily track each mealybug during the behavioral assays, each insect position was noted on a separate paper sheet before the experiment, and further position changes were noted by an observer [16]. To reduce the potential influence of visual cues surrounding the testing arena on the insect behavior, the observer was dressed in a white coat, and the arena was surrounded by a white wall of filter paper (Whatman no. 1, height 30 cm) [17].

After introducing the parasitoid in the experimental arena, the occurrence and duration (s) of the following displays were noted and used to construct an ethogram: (i) latency (i.e., the time spent by *A. vladimiri* remaining stationary before starting host searching), (ii) host searching (i.e., the parasitoid walks around performing antennal tapping), (iii) host encounter (i.e., the parasitoid detects a potential host and stops close to it), (iv) antennal examination (i.e., the parasitoid remains still and performs antennal tapping on the host), (v) ovipositor probing (i.e., the parasitoid swiftly inserts its ovipositor in the host, if the event lasts more than 10 s, an oviposition succeeds; Benelli G., personal observation), (vi) and oviposition (i.e., the parasitoid keeps the ovipositor inside the host’s body, laying an egg) [16]. (vii) Host dragging during oviposition (i.e., the host moves away dragging the ovipositing parasitoid around), as well as (vii) host defensive displays (i.e., the mealybug performs quick body movements against the parasitoid), were also recorded. The possible occurrence and duration of host feeding behavior was noted, following Bokonon-Ganta et al. [18]. The number of mealybugs encountered, examined, and probed by each parasitic wasp within the observation period was noted [19]. The observation period was 20 min; *A. vladimiri* females not starting any of the host-seeking displays detailed above within 10 min were discarded from the study [16]. Each no-choice observation was replicated 50 times.

Furthermore, we assessed the host suitability of *P*. *comstocki* young females for the development and survival of *A*. *vladimiri,* and the quality of progeny. Both in the *P*. *comstocki* and *P*. *ficus* no-choice experiments, the parasitoid was left in the arena for 24 h after the 20-min observation period, allowing the female to parasitize mealybugs. Then, the exposed hosts were monitored daily for 30 days using a stereomicroscope, noting the parasitism rate (i.e., % of mummified mealybugs) and development duration (i.e., the period between *A. vladimiri* parasitization and adult emergence). The number and sex ratio of *A*. *vladimiri* offspring on the two hosts were also determined [16].

Hind tibia length was used as a surrogate for body length [19]. Newly emerged parasitoids were stored in 70% ethanol 24 h after their emergence. Their left hind tibia was removed and mounted on a microscope slide. The left hind tibia length of all adult parasitoids was measured with an ocular micrometer at 60× to investigate the effect of host species on the fitness of parasitoids [16].

#### 2.2.2. Two-Choice Tests

In the two-choice tests, a single *A*. *vladimiri* female was exposed to a mixed population of *P*. *comstocki* and *P*. *ficus* young females, transferring 4 individuals per species into the experimental arena. The *P*. *comstocki* and *P*. *ficus* young females were alternately distributed close to the borders of the area, equally distanced each other. The *A*. *vladimiri* female was released in the center of the testing arena, equally distanced from all the mealybug individuals.

Following the method reported above for “No-choice tests”, after the introduction of an *A. vladimiri* female into the arena, the parasitoid was visually tracked by an observer, and the following parameters were noted: (i) the *A*. *vladimiri* first choice (i.e., which mealybug was first approached with a successful parasitization during the observation time), (ii) the number of successfully parasitized hosts within the observation time, and (iii) the oviposition duration on the selected host (if multiple oviposition acts occurred, only the duration of the oviposition following the first choice on a given host was noted). For each replicate, the observation time was 20 min; 50 replicates were carried out. *A. vladimiri* females not starting any of the host-seeking displays within 10 min were removed from the study [16].

Furthermore, in each replicate *A. vladimiri* was left in the arena for 24 h after the 20 min observations period direct observation, allowing the parasitization of the mealybugs of both species. The successful parasitization of the exposed hosts was monitored daily for 30 days, noting the parasitism rate on *P. comstocki* and *P. ficus* (i.e., % of mummified mealybugs).

### 2.3. Statistical Analysis

In no-choice behavioral assays, differences in the duration of the following behavioral displays, i.e., latency, host searching, antennal tapping, probing and oviposition on the two hosts were evaluated by a General Linear Mixed Model (GLMM) with one factor [20]: y_iw_ = μ + H_i_ + ID_w_ + e_iw_, in which y_iw_ is the observation, μ is the overall mean, H_i_ is the *i*-th fixed effect of the tested mealybug host (*i* = 1–2), ID_w_ is the *w*-th random effect of the parasitoid over repeated host searching and parasitization events (*w* = 1–50) and e_iw_ the residual error; *p* < 0.05 was used to assess significance of differences between means.

Differences in the number of parasitoids displaying host encounter, antennal tapping, probing, oviposition, host dragging, kicking and superparasitization on the two mealybug hosts and parasitoid emergence were evaluated using the Kruskal–Wallis test (*p* = 0.05), while parasitization rates were evaluated using the Wilcoxon test (*p* = 0.05).

Differences in the hind tibia length of parasitoids emerged from the two hosts were evaluated using a weighted generalized linear model (GLZ, Poisson distribution) with one fixed factor [21]: *y* = *Xß* + *ε* where *y* is the vector of the observations (the hind tibia length), *X* is the incidence matrix, *ß* is the vector of the fixed effect (the mealybug host) and ε is the vector of the random residual effects.

In two-choice tests, differences between the number of *A*. *vladimiri* first choices when parasitizing *P*. *comstocki* vs. *P. ficus* were evaluated using a likelihood ratio *χ*^2^ tests, with Yates’ correction [22]. Furthermore, differences in the number of successfully parasitized hosts within 20 min and 24 h on the two mealybug hosts were analyzed with a Wilcoxon test (*p* = 0.05). Differences in the *A. vladimiri* oviposition duration on the two mealybug hosts were evaluated using the Kruskal–Wallis test (*p* = 0.05). JMP 9 (SAS) was used for all the analyses.

## 3. Results

### 3.1. No-Choice Tests

**Figure 1:**
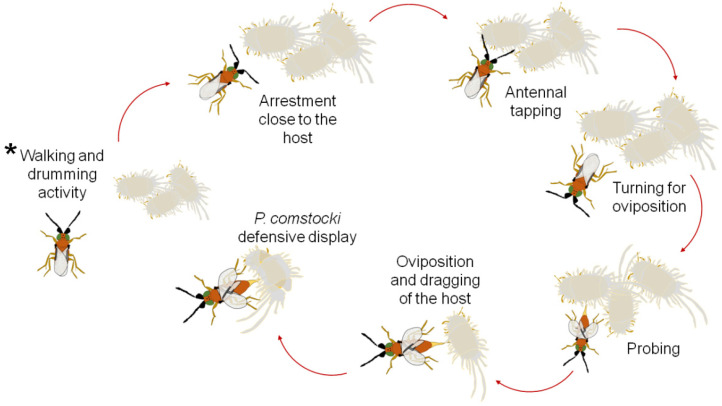
Host searching and parasitization behavior of *Anagyrus vladimiri* towards *Pseudococcus comstocki* (the asterisk indicates where the behavioral sequence begins).

In no-choice tests, when *A. vladimiri* encountered a potential host, it started antennal tapping on the mealybug body to accept or reject the host (Figure 1). If the host was of interest, *A. vladimiri* turned itself, everted the ovipositor and attempted to probe.

**Figure 2:**
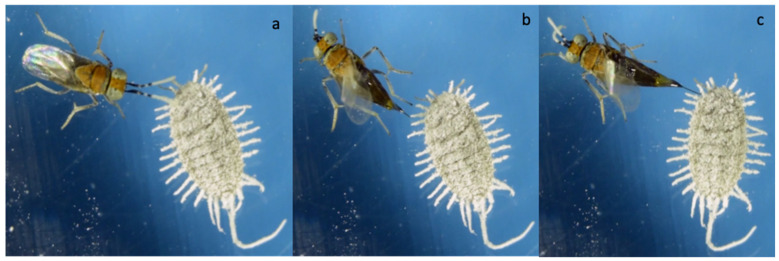
Key behavioral displays performed by *Anagyrus vladimiri* during host searching and parasitization on *Pseudococcus comstocki*: (**a**) antennal tapping, (**b**) probing, and (**c**) oviposition.

If the host was not suitable, the parasitoid moved away and started again the host-seeking activity. Oviposition occurred after a positive probing outcome, usually on the dorso-lateral side of the host (Figure 2).

Overall, the displays composing the host-seeking and parasitization behavior of *A*. *vladimiri* on *P*. *comstocki* were walking and drumming activity, arrestment close to the host, antennal tapping, probing, oviposition, and host dragging. In addition, a peculiar host defensive behavior was noted, i.e., a fast abdominal rocking movement against the parasitoid (kicking), coupled or not with the production of a viscous secretion against the parasitoid to impair its wings [23,24]. Host feeding behavior was not observed.

**Figure 3:**
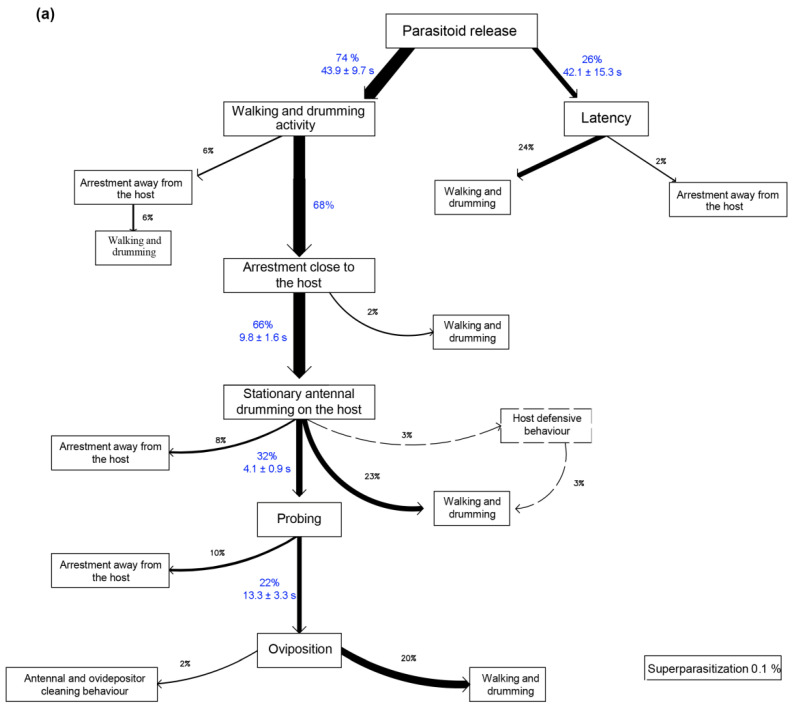

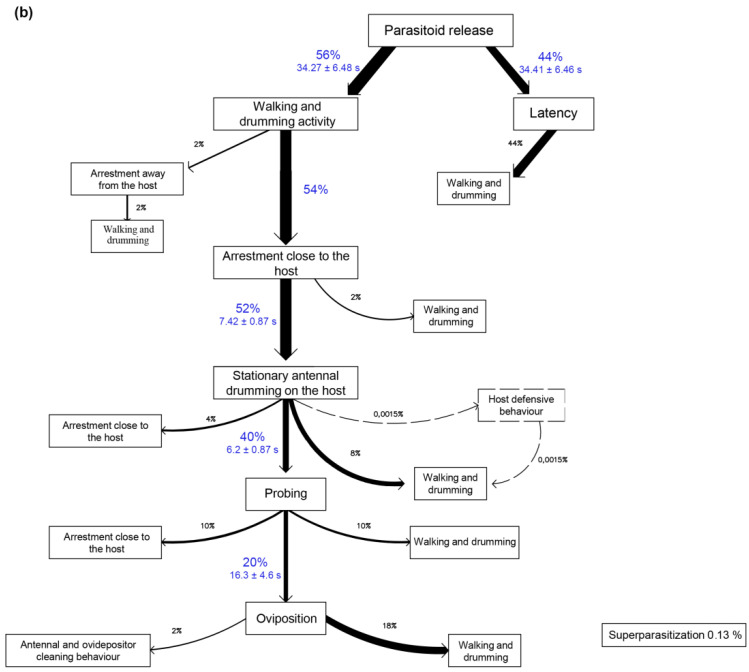
Quantitative analysis of behavioral displays performed by *Anagyrus vladimiri* parasitizing *Pseudococcus comstocki* (**a**) and *Planococcus ficus* (**b**). (% values indicate the percentage of individuals performing a given display out of the total number of individuals in the experiment; when it was possible to quantify it, the duration of each display is provided in seconds (s) (means ± SD); Superparasitism (%) of each host is provided in the lower right corner of each figure).

The ethograms of *A. vladimiri* parasitizing *P. comstocki* (Figure 3a) and *P. ficus* (Figure 3b) were built analyzing the first host-seeking and oviposition event observed in fifty *A. vladimiri* females.

As detailed in Figure 3, *A*. *vladimiri* showed comparable host seeking and oviposition sequences towards both mealybug hosts. However, in our no-choice tests, *A*. *vladimiri* detected a slightly higher number of *P. comstocki* individuals over *P*. *ficus* (68% vs. 54%, respectively) although this does not mean that one was detected more than the other. Most of the selected hosts were subjected to antennal examination, lasting less than 10 s on both hosts. Probing was observed in 32% of parasitoids attacking *P*. *comstocki*, while probing on *P*. *ficus* was 40%. The oviposition rate was 22% on *P. comstocki* and 20% on *P*. *ficus*. The above-described host defensive behavior showed by *P*. *comstocki* and *P. ficus* against *A*. *vladimiri* was observed in 3% and 0.0015% of the parasitoid-host interactions, respectively. *A*. *vladimiri* superparasitization acts occurred on both hosts with a comparable rate (*P*. *comstocki* 0.1% and *P*. *ficus* 0.13%).

**Figure 4:**
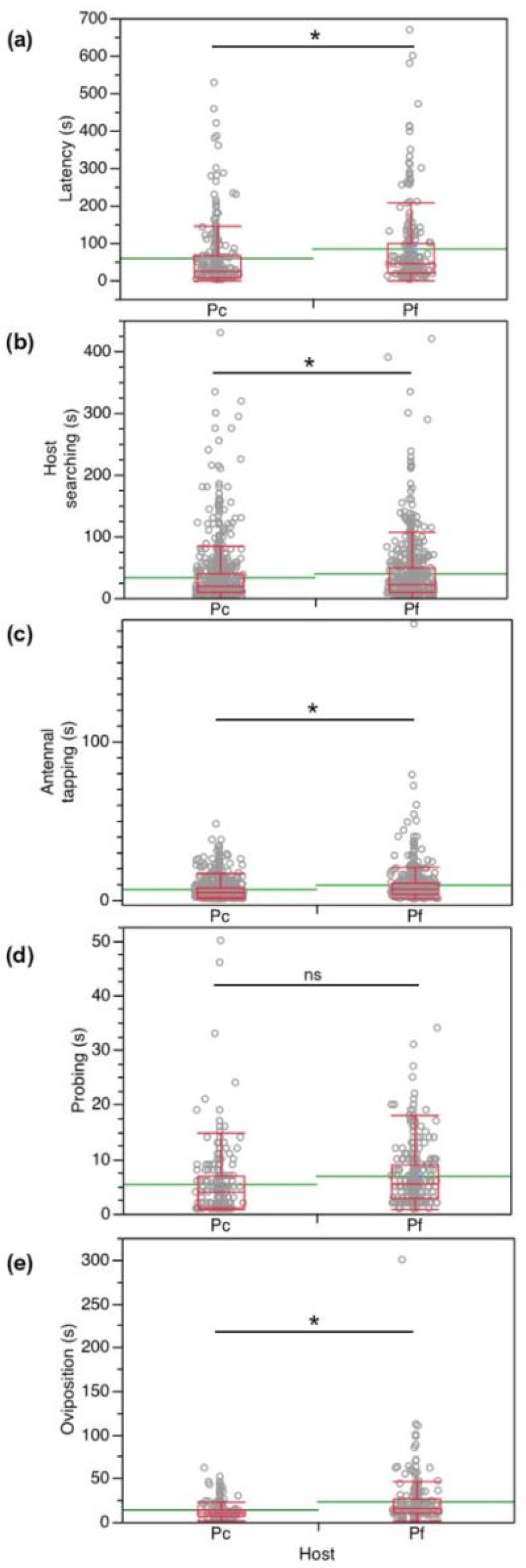
Time spent by *Anagyrus vladimiri* during (**a**) latency, (**b**) host searching, (**c**) antennal tapping, (**d**) probing, and (**e**) oviposition displays in no-choice tests carried out on *Pseudococcus comstocki* (Pc) and *Planococcus ficus* (Pf). Each box plot indicates the median (central line) and its range of dispersion (lower and upper quartiles and outliers); green lines indicate the means. Asterisks indicate significant differences (GLM, *p* < 0.05); ns = not significant.

To understand the parasitization efficiency of *A. vladimiri* towards *P. comstocki* and *P. ficus*, the above-mentioned behavioral displays were compared in terms of duration (s) and frequency (no. of acts). Concerning duration, results showed a significant difference for latency (*F_1,58_* = 6.71, *p* = 0.0120), host searching (*F_1,73_* = 3.9, *p* = 0.05), antennal tapping (*F_1,84_* = 18.9, *p* < 0.0001) and oviposition (*F_1,107_* = 3.75, *p* < 0.0001). As a general trend, the duration of these displays was longer in *P. ficus* than in *P. comstocki*. There was no significant difference in probing behavior between the two species (*F_1,93_* = 3.75, *p* = 0.056) (Figure 4).

**Figure 5:**
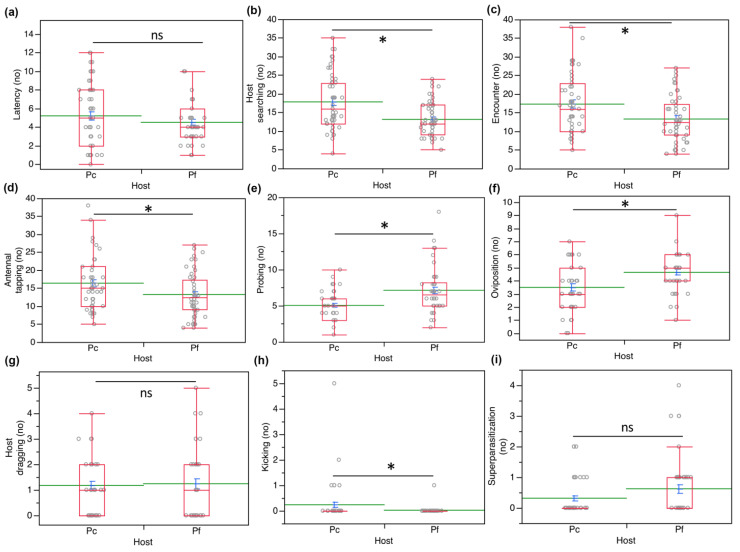
Frequency (no.) of different behavioral parameters performed by *Anagyrus vladimiri* approaching *Pseudococcus comstocki* (Pc) and *Planococcus ficus* (Pf) in no-choice tests: (**a**) latency, (**b**) host searching, (**c**) host encounter, (**d**) antennal tapping, (**e**) probing, (**f**) oviposition, (**g**) host dragging, (**h**) kicking (fast abdominal rocking movement against the parasitoid), and (**i**) superparasitization. Each box plot indicates the median (central line) and its range of dispersion (lower and upper quartiles and outliers); green lines indicate the means, while light blue T-bars indicate standard errors. Asterisks indicate significant differences (Kruskal–Wallis test, *p* < 0.05), ns = not significant.

The frequency of behavioral displays performed by *A*. *vladimiri* approaching its host in no-choice tests highlighted some differences about host searching (*χ^2^* = 11.17, *d.f.* *=* 1, *p* = 0.0008), encounter (*χ^2^* = 6.31, *d.f.* = 1, *p* = 0.012), antennal tapping (*χ^2^* = 4.38, *d.f.* *=* 1, *p* = 0.0362), probing (*χ^2^* = 12.4, *d.f. =* 1, *p* = 0.0004), oviposition (*χ^2^* = 9.95, *d.f.* *=* 1, *p* = 0.0016) and host defensive behavior (*χ^2^* = 4.77, *d.f.* *=* 1, *p =* 0.029). However, comparing latency (*χ^2^* *=* 0.86, *d.f.* *=* 1, *p* = 0.35), host dragging (*χ^2^* *=* 0.0085, *d.f.* = 1, *p* = 0.92) and superparasitization (*χ^2^* = 3.15, *d.f.* *=* 1, *p* = 0.076), the results showed no significant differences between *P*. *comstocki* and *P*. *ficus* (Figure 5).

**Figure 6:**
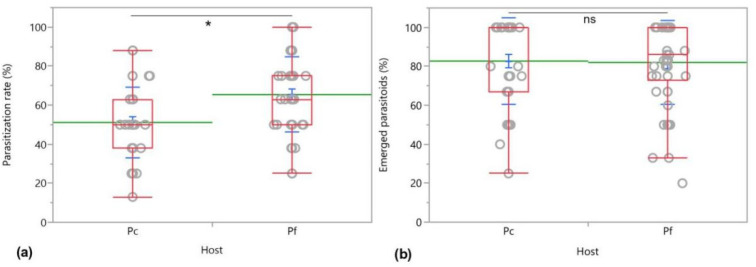
Parasitization (**a**) and emergence rates (%) (**b**) of *Anagyrus vladimiri* on *Pseudococcus comstocki* (Pc) and *Planococcus ficus* (Pf) in no-choice tests. Parasitization rate were analyzed with Wilcoxon test (*p* < 0.05) while emerged parasitoids with Kruskal–Wallis test (*p* < 0.05). The asterisk indicates significant difference between parasitoids emerged from the two hosts, ns = not significant.

The parasitization rate, percentage of emerged parasitoids, developmental time and sex ratio of *A*. *vladimiri* developed on *P*. *comstocki* were compared with those from wasps developed on *P*. *ficus* (Figure 6). Significant differences between the percentage of parasitized hosts were found (*χ^2^* = 11.808, *d.f.* = 1, *p =* 0.0006) (Figure 6). The number of parasitoids emerged from the two hosts was not statistically different (*χ^2^* = 0.141, *d.f. =* 1, *p* = 0.707).

**Figure 7:**
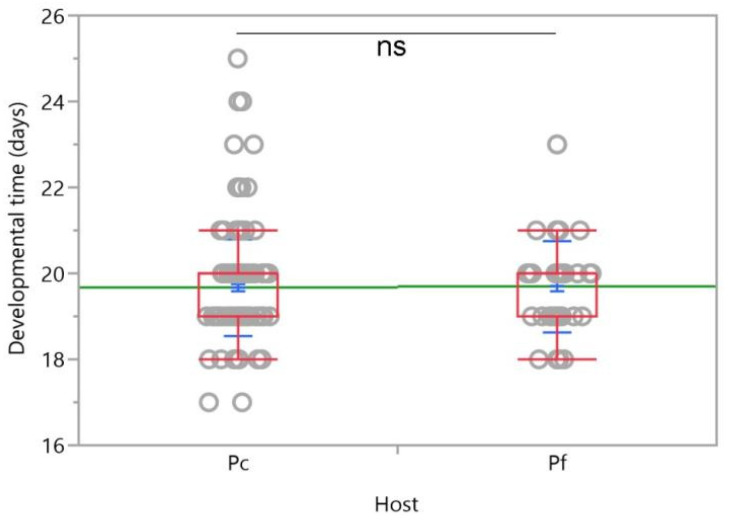
Developmental time of *Anagyrus vladimiri* from *Pseudococcus comstocki* (Pc) and *Planococcus ficus* (Pf). Each box plot indicates the median (central line) and its range of dispersion (lower and upper quartiles and outliers); green lines indicate the means, while light blue T-bars indicate standard errors; ns = not significant difference in development time between parasitoids emerged from the two hosts (Kruskal–Wallis test, *p* > 0.05).

On the other hand, no significant difference in the developmental time of the offspring of *A*. *vladimiri* from the hosts was found (*χ^2^* *=* 0.088, *d.f. =* 1, *p* = 0.7675) (Figure 7). The first adult emerged after 17 days from *P. comstocki* (with a mean emergence time of 19.67 ± 1.12 (mean ± SD)) and 18 days from *P*. *ficus* (with a mean emergence time of 19.70 ± 1.07 (mean ± SD)). Moreover, no difference in the sex ratio was noted between parasitoid progeny emerging from *P*. *comstocki* and *P*. *ficus* (adult sex ratio (ASR): 1.16 and 1.58 for female *P*. *comstocki* and *P*. *ficus*, respectively).

**Figure 8:**
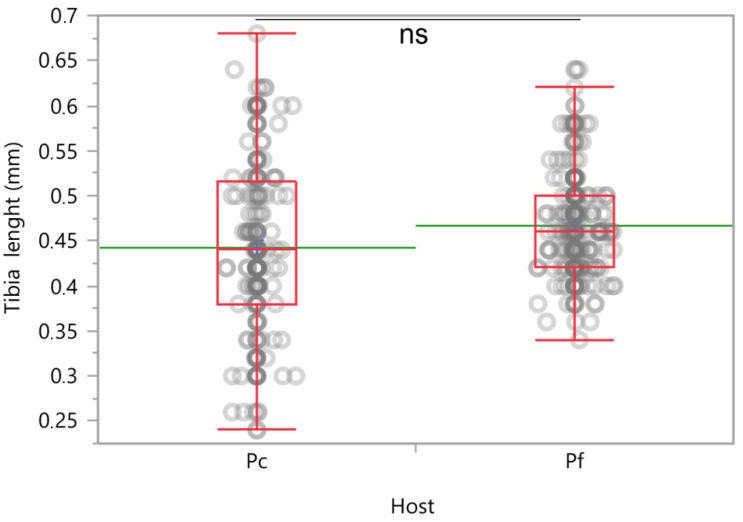
Hind tibia length of *Anagyrus vladimiri* emerged from *Pseudococcus comstocki* (Pc) and *Planococcus ficus* (Pf). Each box plot indicates the median (central line) and its range of dispersion (lower and upper quartiles and outliers); green lines indicate the means; ns = no significant difference in tibia length of parasitoids emerged from different hosts (GLZ, *p* > 0.05).

The length of the hind tibia of emerged *A. vladimiri* was used as a surrogate of body length to evaluate the fitness of the offspring [19]. No significant difference was found between the two hosts (*χ^2^* = 0.12, *d.f.* = 1, *p* = 0.720) (Figure 8), confirming that *P. comstocki* is a highly suitable host for *A. vladimiri*.

### 3.2. Two-Choice Tests

**Figure 9:**
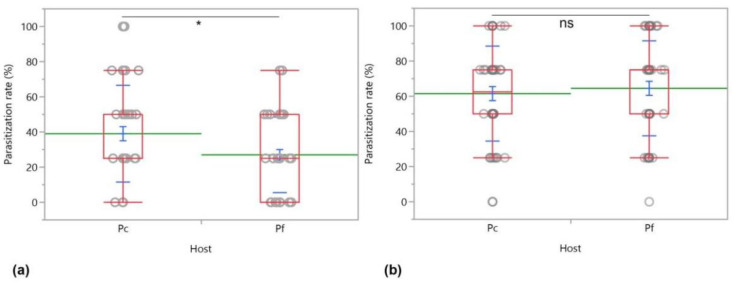
Parasitization rates of *Anagyrus vladimiri* on *Pseudococcus comstocki* (Pc) and *Planococcus ficus* (Pf) during a two-choice direct observation lasting 20 min (**a**), and after a 24 h-exposure period of the mixed mealybug population (4 + 4 young females) to the parasitoid (**b**). Each box plot indicates the median (central line) and its range of dispersion (lower and upper quartiles, and outliers); green lines indicate the means, while light blue T-bars indicate standard errors. The asterisk indicates a significant difference (Wilcoxon test, *p* < 0.05); ns = not significant.

*Anagyrus vladimiri* did not show a significant preference for *P. comstocki* over *P. ficus* in terms of first parasitization choice (23 vs. 27 first choices on *P. comstocki* and *P. ficus*, respectively, *χ^2^* = 0.340, *d.f. =* 1, *p* = 0.560). However, *A. vladimiri* successfully parasitized more *P. comstocki* over *P. ficus* within the observation time (20 min) (*χ^2^* = 4.101, *d.f. =* 1, *p* = 0.042) (Figure 9a), but this difference was not confirmed after a 24 h exposure period of *A. vladimiri* to the mixed population of both mealybug hosts (*χ^2^* = 0.3440, *d.f. =* 1, *p* = 0.558) (Figure 9b).

**Figure 10:**
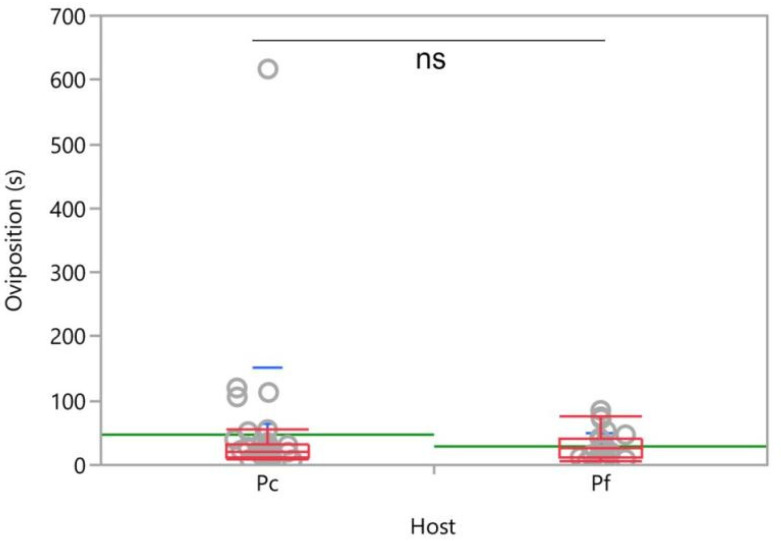
Oviposition duration of *Anagyrus vladimiri* in two-choice 20-min tests evaluating host preferences for *Pseudococcus comstocki* (Pc) vs. *Planococcus ficus* (Pf). Each box plot indicates the median (central line) and its range of dispersion (lower and upper quartiles an and outliers); green lines indicate the means, while light blue T-bars indicate standard errors; ns = not significant, it indicates the lack of significant differences (Kruskal–Wallis test, *p* > 0.05).

The oviposition duration of *A. vladimiri* on *P. comstocki* was not different from that recorded on *P. ficus* (*χ^2^* = 0.002, *d.f. =* 1, *p* = 0.964) (Figure 10).

## 4. Discussion

This study aimed to understand whether *A. vladimiri* could be used to effectively manage *P. comstocki* based on the knowledge that this wasp is a reliable parasitoid of *P. ficus* [12,25]. However, there was no evidence about the suitability of *P. comstocki* as a host for *A. vladimiri*, despite the growing importance of Comstock mealybugs [2]. The present study proved the successful development of *A. vladimiri* on *P. comstocki*. An accurate comparison between the two hosts was provided by the parasitization rate and supported by the quantification of frequencies and durations of the characteristic behaviors involved in the host location and parasitization sequence (i.e., host searching, encounter, antennal tapping, probing, oviposition, host dragging, kicking, and superparasitization). Although some displays were more frequent or prolonged on one host species over the other, the parasitization rates as well as the percentage of parasitoids emerged from the respective hosts, were fully comparable. Earlier research on closely related *Anagyrus* species showed comparable parasitization rates on different hosts of agricultural importance. For example, testing *Anagyrus kamali* Moursi (Hymenoptera: Encyrtidae) on *Maconellicoccus hirsutus* (Green) (Hemiptera: Pseudococcidae), [26] a parasitization rate of 65% was found, while *Aenasius bambawalei* Hayat (Hymenoptera: Encyrtidae) parasitized 52% of *Phenacoccus solenopsis* Tinsley (Hemiptera: Pseudococcidae) [27]. However, concerning the emergence rate, Sagarra and Vincent [26] found much lower values than ours (between 19% and 48.5% depending on the age of the host), while Zhang et al. [27] obtained an emergence rate (81.78%) similar to our results. Of note, in our study, the number of males emerged from *P. ficus* was higher than females.

A key feature when assessing host suitability is the evaluation of the fitness of the parasitoid progeny. As stressed by Sagarra et al. [19], body length and hind tibia length are linearly related, thus the hind tibia length can be a precise and rapid tool to evaluate the overall size of the parasitoid and, therefore, its fitness. In our results, the tibia length of *A. vladimiri* progeny emerged from the two host species was not statistically different, showing that the fitness of the newly emerged parasitoids was not compromised when *P. comstocki* was exploited as a host. Of note, the two-choice tests carried out by introducing mated *A. vladimiri* in the arena with a mixed population of four *P. comstocki* and four *P. ficus* young females revealed no preferences for a particular host, thus confirming that the parasitoid accepted both hosts. The parasitization rate of the two species after 24 h of host exposure to the female wasp was similar as well.

The capability of *A. vladimiri* to successfully parasitize different species of invasive mealybugs makes it a highly adaptable BCA. On the other hand, this may represent a relative risk of high likelihood of non-target effects if the insect needs to be introduced in regions where it is not native [28,29]. However, since *A. vladimiri* is naturally present in many fruit-producing regions worldwide [11,12], mass releases may boost the local population of the parasitoid, positively enhancing IPM and biocontrol programs.

This is confirmed by our results, as well as by other papers where this encyrtid has been evaluated against various mealybugs, e.g., *P. ficus*, *Planococcus citri* (Risso), *Pseudococcus calceolariae* (Maskell), *Pseudococcus viburni* (Signoret), and *Phenacoccus peruvianus* Granara de Willink (Hemiptera: Pseudococcidae) [30]. For instance, in this study, it is shown that *A. vladimiri* can complete its development on all the hosts although with different success rates. Further tests have shown significant differences in the behavioral patterns of host recognition, host handling, and the level of host acceptance [31]. In our research, the lack of differences in the sequence of events leading to oviposition, the main behavioral parameters as well as to the parasitization success in all the performed tests, support the use of *A. vladimiri* as effective BCA for *P. comstocki* management. Moreover, the utilization of both species suggests that *A. vladimiri* can be released to manage the infestation of a single mealybug pest, as well as in scenarios where both mealybug species are present simultaneously. This is a common situation in many fruit orchards of North and Central Italy (G. Benelli, pers. observ.).

## 5. Conclusions

Our no-choice experiments showed several differences in the frequency and duration of selected displays characterizing the host-seeking and oviposition of *A*. *vladimiri* on *P*. *comstocki* and *P*. *ficus*. However, both mealybug species were equally suitable as hosts for *A. vladimiri* and supported the production of progeny with similar body size. Furthermore, the results from two-choice tests highlighted that *P*. *comstocki* was preferred by *A*. *vladimiri* females in a comparable manner to its classic host *P. ficus*. Overall, our findings showed that *A*. *vladimiri* successfully parasitized and developed on *P*. *comstocki*, therefore, highlighting that this encyrtid species may have general utility in biological control programs with one or more mealybug species.

## Data Availability

Data are contained within the article.
